# Science of health care delivery milestones for undergraduate medical education

**DOI:** 10.1186/s12909-017-0986-0

**Published:** 2017-08-25

**Authors:** Rachel D. Havyer, Suzanne M. Norby, Andrea N. Leep Hunderfund, Stephanie R. Starr, Tara R. Lang, Alexandra P. Wolanskyj, Darcy A. Reed

**Affiliations:** 1 Mayo Clinic, 200 First St. SW, Rochester, MN 55905 USA; 2 Mayo Clinic School of Medicine, Mayo Clinic, Rochester, MN USA; 3 Gundersen Health System, LaCrosse, WI, 1900 South Avenue, La Crosse, WI 54601 USA

**Keywords:** Science of health care delivery, Milestones, Undergraduate medical education, Assessment

## Abstract

**Background:**

The changing healthcare landscape requires physicians to develop new knowledge and skills such as high-value care, systems improvement, population health, and team-based care, which together may be referred to as the Science of Health Care Delivery (SHCD). To engender public trust and confidence, educators must be able to meaningfully assess physicians’ abilities in SHCD. We aimed to develop a novel set of SHCD milestones based on published Accreditation Council for Graduate Medical Education (ACGME) milestones that can be used by medical schools to assess medical students’ competence in SHCD.

**Methods:**

We reviewed all ACGME milestones for 25 specialties available in September 2013. We used an iterative, qualitative process to group the ACGME milestones into SHCD content domains, from which SHCD milestones were derived. The SHCD milestones were categorized within the current ACGME core competencies and were also mapped to Association of American Medical Colleges’ Entrustable Professional Activities (AAMC EPAs).

**Results:**

Fifteen SHCD sub-competencies and corresponding milestones are provided, grouped within ACGME core competencies and mapped to multiple AAMC EPAs.

**Conclusions:**

This novel set of milestones, grounded within the existing ACGME competencies, defines fundamental expectations within SHCD that can be used and adapted by medical schools in the assessment of medical students in this emerging curricular area. These milestones provide a blueprint for SHCD content and assessment as ongoing revisions to milestones and curricula occur.

**Electronic supplementary material:**

The online version of this article (10.1186/s12909-017-0986-0) contains supplementary material, which is available to authorized users.

## Background

Current and future physicians must acquire and maintain a broad array of knowledge and skills to maximize health outcomes, many of which are not included in formal curricula or accreditation requirements. Historically, complexities of modern health care systems have not been addressed during traditional medical training, and physicians are not adequately prepared to practice within and improve complex health care systems [1]. With the rapid change and reform in health care delivery today, physicians need to learn new knowledge and skills [2] in order to help achieve the Triple Aim outlined by the Institute for Healthcare Improvement [3]. To address these gaps, physician education in novel domains within the Accreditation Council for Graduate Medical Education (ACGME) Systems-Based Practice and Practice-Based Learning and Improvement competencies is needed [4–7]. The content of these novel domains is broad and includes topics such as systems improvement, clinical informatics, high-value care, systems and human factors engineering, health policy, and population health [4, 8]. Taken together, this content has been referred to as the third science [8], healthcare delivery science [9], health systems science [10], and herein, the science of health care delivery (SHCD) [11]. Although the terms vary, all refer to a new set of physician capabilities essential for practice that extend beyond the basic sciences and clinical skills traditionally taught in medical schools [12].

In order to engender public trust and confidence in physicians’ abilities, educators must be able to meaningfully measure physicians’ capabilities in SHCD. The ACGME has adopted milestones for measuring competence in graduate medical education (GME) [13], and the Association of American Medical Colleges (AAMC) has defined core Entrustable Professional Activities (EPAs) to ensure that medical students are prepared for residency training [14, 15].

With this in mind, we aimed to develop a set of SHCD milestones based on published ACGME milestones and corresponding to AAMC EPAs that can be used by medical schools to determine students’ competence in SHCD. These milestones define fundamental expectations within SHCD that are needed for all physicians to help optimize patients’ health outcomes within complex modern healthcare systems. The SHCD milestones may provide a blueprint for SHCD content and assessment, and inform future revisions to ACGME milestones. The authors report no competing interests.

## Methods

We reviewed key references and consulted with experts in the areas of health systems, quality, and medical education to identify core topics related to the SHCD [2, 4, 16–22]. These topics included patient-centered care (individualized care, cultural competence, shared decision making, health behavior change), population-oriented care (public and population health, advocacy), high-value care (quality, safety, cost, health systems, practice improvement, information technology, clinical informatics), team-based care (inter-professional practice, care transitions), and healthcare policy, law, and regulation.

We reviewed the “Milestone Project” documents available on the ACGME Next Accreditation System website on September 11, 2013 for all 25 available specialties [23]. These documents included 601 sub-competencies and 6667 individual milestone elements, which served as our unit of analysis. Each milestone element was reviewed by two authors, and elements that were felt to be related to the key SHCD topics by one or more of the authors were identified. A third author then reviewed and resolved any discrepancies with additional input from the group if uncertainties remained. All included milestone elements were then sorted via group discussion into subsets sharing a common theme. These themes informed the subsequent definition of SHCD sub-competencies which were categorized within the existing six ACGME core competencies based on established descriptions in the ACGME Common Program Requirements [24]. If SHCD milestones mapped to more than one ACGME competency, the most relevant competency was selected.

The SHCD milestones were also mapped to AAMC Core EPAs based on descriptions provided in the AAMC Core EPAs Curriculum Developers’ Guide [14]. Select ACGME milestone elements were incorporated into the SHCD milestones as appropriate. Milestone elements appropriate for an early UME level (e.g. ‘novice’ level) that did not exist in the ACGME milestones documents were developed. Finally, additional milestones were created to supplement SHCD topics that were missing or insufficiently robust in the ACGME milestones.

## Results

The resulting set of SHCD milestones is provided in Additional file 1. Each includes references to representative ACGME milestones upon which the SHCD milestones were based. Table 1 shows the categorization of the 15 sub-competencies for these SHCD milestones within the existing ACGME core competencies, along with corresponding AAMC EPAs.

**Table 1:** Sub-competencies for science of health care delivery milestones in undergraduate medical education

ACGME core competency [23]	Sub-competencies for SHCD milestones	Related AAMC core EPA functions or behaviors [14]
Systems-Based Practice	Develops skills in advocacy theory, execution and communication in order to advocate effectively for individual patients and at-risk populations	• Advocates for patient access to community resources (EPA 9)
	Partners with the community to improve individual and population health	• Understands that population health issues affect the health of patients and therefore identifies sources of information about the needs and resources of the community; Interacts and begins to work collaboratively with community agencies, professionals, and others to address population health issues (EPA 3 and 7)
	Defines value in health care and applies high value care strategies for individual patients and populations	• Incorporate cost awareness and principles of cost-effectiveness and pre-test/post-test probability in developing diagnostic plans (EPA 3)
	Identifies systems failures and errors and contributes to a culture of safety and quality improvement	• Recognize and avoid errors by using safety alerts (e.g., drug-drug interactions) and information resources to place the correct order and maximize therapeutic benefit and safety for patients (EPA 4) • Identify system failures and contribute to a culture of safety and improvement (EPA 13)
	Describes United States’ healthcare financing and related effects on patient care and quality	• None
	Analyzes current United States’ healthcare policy and its impact on health care delivery systems	• None
	Describes the roles of clinical informatics, healthcare IT, and technology assessment in improving patient outcomes	• Uses tools and information technology to support decision making and adopt strategies to decrease cost and risk to individuals (EPA 3) • Compose orders efficiently and effectively, such as by identifying the correct admission order set, selecting the correct fluid and electrolyte replacement orders, and recognizing the needs for deviations from standard order sets (EPA 4) • Identify and demonstrate the use of information technology to access accurate and reliable online medical information (EPA 7) • Document, and update, an electronic handover tool (EPA 8)
Interpersonal and Communication Skills	Develops patient-centered perspective and interviewing skills	• Demonstrate patient-centered interview skills (EPA 1) • Apply the primary findings of one’s information search to an individual patient or panel of patients (EPA 7)
	Collaborates as a member of an inter-professional team and demonstrates effective, team-based patient care	• Demonstrates an understanding of the roles of various team members by interacting with appropriate team members to accomplish assignments. Actively works to integrate into team function and meet or exceed the expectations of his or her given role (EPA 9)
	Effectively leads an intra−/ inter-professional team in the clinical, educational, or research setting	• Collaborate as a member of an inter-professional team (EPA 9)
	Effectively gives and receives a patient handover to transition care responsibility	• Give or receive a patient handover to transition care responsibility (EPA 8)
Professionalism	Individualizes care by engaging patients in shared decision making and motivating behavior change	• Elicit and take into account patient preferences in making recommendations (EPA 3) • Discuss the planned orders and prescriptions… with patients and families and use a nonjudgmental approach to elicit health beliefs (EPA 4) • Document patient preferences to allow their incorporation into clinical decision making (EPA 5)
	Practices contextual and cultural humility, curiosity, and awareness	• Consider cultural and other factors that may influence the patient’s description of symptoms; Demonstrate cultural awareness and humility (EPA 1)
Practice-Based Learning and Improvement	Forms clinical questions and retrieves, appraises and assimilates evidence from the scientific literature	• Form clinical questions and retrieve evidence to advance patient care (EPA 7)
Patient Care	Applies principles of preventive health and strategies for population health management	• Understands that population health issues affect the health of patients and therefore identifies sources of information about the needs and resources of the community (EPA 3 and 7) • Apply the primary findings of one’s information search to an individual patient or panel of patients (EPA 7)

*Abbreviations*: *AAMC* Association of American Medical Colleges; *ACGME* Accreditation Council for Graduate Medical Education; *EPA* Entrustable Professional Activities; *IT* Information Technology; *SHCD* Science of Health Care Delivery

The SHCD milestones are organized within 5 levels, similar to most ACGME specialty milestones [23]. Level 1 milestone elements describe a novice level student and level 5 elements describe aspirational levels of performance typically achieved during residency or beyond.

Table 2 provides an example of the SHCD milestones related to shared decision making and motivating behavior change. Shared decision making is the process of understanding the patient’s values, exchanging information about available options and their consequences, and coming to a decision based on consensus [25]. The aforementioned sub-competency incorporates these components of shared decision making between levels 1 to 5 by 1) supporting patient autonomy and eliciting patient values (“Uses directive style of guiding patient decisions” to “Develops care plans jointly with the patients and caregivers”); 2) utilizing appropriate communication strategies (“Uses technical terms and jargon” to “Consistently demonstrates communication strategies to ensure patient understanding and matches communication modality to the patient needs, health literary and context”); and 3) utilizing tools to assist in decision making and health behavior change (“Conducts interview without inquiring about social and behavioral factors that affect the health of individuals” to “Adapts decision aids and health behavior coaching techniques for patients who are not motivated to change”). The terms used to describe the target knowledge/skill level within these milestones progress according to Bloom’s taxonomy [26], with lower-level objectives at the critical deficiency and novice stages (e.g. ‘awareness’, ‘identify’) and higher-level objectives at the competent and aspirational stages (e.g. ‘demonstrates’, ‘implements’, ‘adapts’).

**Table 2:** Example of one of the 15 science of health care delivery sub-competencies with associated milestones

Individualizes care by engaging patients in shared decision making and motivating behavior change				
Milestones				
Level 1	Level 2	Level 3	Level 4	Level 5
Uses directive style of guiding patient decisions	Respects patient autonomy in healthcare decisions Discusses potential challenges associated with integrating patient values and beliefs with those of self, society, and the core values of medicine	Recognizes opportunities for shared decision making	Engages patients in shared decision making	Develops care plans jointly with patients and caregivers
Uses technical terms and jargon		Uses easy-to-understand language in all phases of communication	Assesses patient understanding of health information and invites questions	Consistently demonstrates communication strategies to ensure patient understanding and matches communication modality to the patient needs, health literacy and context
Conducts interview without inquiring about social and behavioral factors that affect the health of individuals	Identifies social and behavioral factors that affect the health of individuals and may be modifiable	Describes effective approaches to modifying individual health behaviors, such as shared decision making aids, motivational interviewing and health behavior coaching	Implements decision aids, motivational interviewing and/or health behavior coaching to modify individual health behaviors for patients who are motivated to change	Adapts decision aids and health behavior coaching techniques for patients who are not motivated to change
^a^Representative Corresponding ACGME Milestones [23]				
Derm ICS1	Aero Med MK1, FM ICS1, TY Prof2, Occ Med MK1	Aero Med MK1, Occ Med MK1, Prev MK1, Psych ICS2, Derm ICS1, FM ICS2	Aero Med MK1, FM ICS2, Oph ICS1, Occ Med MK1, Prev MK1, Psych ICS2, Uro ICS2, IM ICS1	Aero Med MK1, FM ICS2, Neuro ICS, Occ Med MK1, Peds SBP1, Prev MK1, Psych ICS2 & Prof1,PS Prof1

^a^Referenced milestones are representative, not comprehensive. Reference Milestone Abbreviations: *GME* Graduate medical education, *ICS* interpersonal and communication skills, *MK* medical knowledge, *Prof* professionalism, *SBP* systems-based practice. Specialty Abbreviations: *Aero Med* aerospace medicine, *FM* family medicine, *Neuro* neurology, *Occ Med* occupational medicine, *Oph* ophthalmology, *Peds* pediatrics, *PS* plastic surgery, preventative/public health, *Psych* psychiatry, *TY* transitional year, *Uro* urology

## Discussion

We propose this novel set of SHCD milestones, derived from published ACGME milestones and linked to AAMC EPAs, for use by medical schools to assess students’ knowledge and skills in SHCD. These milestones define fundamental expectations within SHCD and provide a blueprint for SHCD education and assessment that can be adaptable and responsive to ongoing milestone revisions. These milestones may serve as a starting point for implementation, further development, and refinement by medical schools based on the local curriculum, learning environment, and school-specific needs. While a unique SHCD curriculum is being implemented at our institution, these SHCD milestones were intentionally designed to be devoid of institution-specific contexts to enable broad applicability across educational settings. Until there is consensus among educators regarding a minimal set of SHCD student outcomes for UME, medical schools should individually determine whether and how to implement SHCD milestones and where along the spectrum of performance (e.g. from novice to aspirational) their desired graduation target would lie.

During our review of initial ACGME milestones, we identified several SHCD topics that were infrequently or never mentioned. These areas included patient advocacy, motivational interviewing, health behavior coaching, clinical informatics, and data analytics (beyond typical clinical use of electronic health records). As such, we developed new milestones for these topics in order to assist medical schools in measuring student capabilities in these emerging areas. Likewise, some SHCD topics did not readily map to any of the core EPAs for entering residency, such as healthcare costs and policy. We propose that these important topics also be considered for inclusion among ACGME milestones and EPAs as these undergo future revision.

To operationalize these SHCD milestones, medical schools will need to find time and space within their already established curricula to deliver relevant SHCD content as well as identify appropriate settings in which students’ performance in SHCD milestones can be observed and assessed [12]. Rationale for incorporating SHCD content into existing curricula include not only preparing future physicians for the changing landscape of healthcare but also utilizing medical education as a driver for change in order to add value to the healthcare system [2, 6, 7, 27]. In ranking the importance of the gaps in physician competencies, the American Hospital Association report identified that the top physician gaps were in content areas of SHCD [2]. Since formal, comprehensive SHCD curricula have not traditionally been integrated into medical education, faculty experts and content may not exist for many aspects of SHCD. In such cases, content creation and faculty development will be required. Since few tools for assessing SHCD knowledge and skills exist in the medical literature, it will be incumbent upon educators to develop new assessment tools, ideally those that are generalizable and can be easily adapted to different settings, and to collect validity evidence supporting their use.

These SHCD milestones have several limitations. First, we reviewed ACGME milestones at a single point in time. Yet, revisions have occurred over time and ongoing revisions are anticipated [28]. The SHCD milestones included here are not intended to be static, and should be modified over time as experience with their use accrues. Second, our authorship team represents a single institution. However, the SHCD milestones are not specific to our organization and can be adapted for local use. Third, our team was comprised of the medical disciplines of internal medicine, pediatrics, and neurology, but did not include members of other disciplines such as surgical and procedural specialties. Fourth, we utilized all milestones available at the time of study (which included 25 specialties at that time), however milestones have subsequently been developed for the full complement of ACGME accredited specialties. Thus the SHCD milestones may have limited generalizability to the other sub-specialties until undergoing further revision by those sub-specialties. Finally, we recognize that the scope of SHCD represented by these milestones, while not completely comprehensive, is broad, making implementation of the entire set a challenge. Schools may opt to use a smaller set of SHCD milestones based on their individual priorities.

## Conclusions

SHCD is an emerging curricular area within medical education, and a common set of SHCD milestones may help facilitate the successful preparation of medical students for residency and twenty-first century clinical practice. Valid assessments of students’ capabilities in SHCD may offer helpful metrics to determine their preparedness for residency in these domains. As a medical student would not be expected to demonstrate full competency in all domains by graduation, assessment of SHCD milestones will need to continue during GME and beyond. This novel set of SHCD milestones provides a blueprint for SHCD content and assessment, and a starting point for the medical education community as ongoing revisions to milestones and curricula occur within the emerging area of SHCD.

## Additional file

Additional file 1: Science of Health Care Delivery Milestones Developed for Undergraduate Medical Education with Corresponding Representative ACGME Milestones. This file contains the complete set of Science of Health Care Delivery milestones developed for undergraduate medical education. The milestones are grouped into 15 Science of Health Care Delivery sub-competencies based upon content domains and include references to representative ACGME milestones upon which the milestones were based. (DOCX 78 kb)

## Abbreviations

AAMC: Association of American Medical Colleges
ACGME: Accreditation Council of Graduate Medical Education
EPAs: Entrustable Professional Activities
GME: Graduate Medical Education
SHCD: Science of Health Care Delivery
UME: Undergraduate Medical Education
