# Supplementary material for: Science of health care delivery milestones for undergraduate medical education
Source: BMC Med Educ. 2017 Aug 25;17:145. doi: 10.1186/s12909-017-0986-0 (PMC5572071; doi:10.1186/s12909-017-0986-0)
Supplement: Additional file 1: — Science of Health Care Delivery Milestones Developed for Undergraduate Medical Education with Corresponding Representative ACGME Milestones. This file contains the complete set of Science of Health Care Delivery milestones developed for undergraduate medical education. The milestones are grouped into 15 Science of Health Care Delivery sub-competencies based upon content domains and include references to representative ACGME milestones upon which the milestones were based. (DOCX 78 kb) [file 12909_2017_986_MOESM1_ESM.docx]

**ADDITIONAL FILE 1: Science of Health Care Delivery Milestones Developed for Undergraduate Medical Education with Corresponding Representative ACGME Milestones**

| **Develops patient-centered perspective and interviewing skills** | | | | |
| --- | --- | --- | --- | --- |
| **Milestones** | | | | |
| **Level 1** | **Level 2** | **Level 3** | **Level 4** | **Level 5** |
| Focuses excessively on biomedical model of health and disease  Ignores patient preferences  Does not recognize unique characteristics and needs of patients and families | Identifies the potential impact of patient beliefs, values, preferences, expectations, emotions, and practices on health and health behaviors  Recognizes and respects patient beliefs, values, preferences, expectations, and emotions when offered by the patient/caregiver, but does not actively solicit the personal or emotional context of the illness and treatment  Recognizes nonverbal cues and emotions | Describes strategies for eliciting the beliefs, values, preferences, expectations, emotions, and practices of patients and families in the experience of illness and treatment | Elicits beliefs, values, preferences, expectations, emotions, and practices of patients and families in the experience of illness and treatment  Identifies own clinical information needs based, in part, on the beliefs, values, preferences, and practices of each patient  Responds effectively to nonverbal cues and emotions | Proposes care plans that are informed by patients’ beliefs, values, preferences, and practices  Suggests alternate care plans when patients personal decisions/beliefs preclude the use of commonly accepted practices |
| ^a^**Representative Corresponding ACGME Milestones**[23] | | | | |
| IM ICS1 | IM Prof3, Oph ICS1, | PS Prof1, PS ICS | Derm ICS1,IM Prof3, Psych Prof1, FM PBLI2 | Derm ICS1, EM Prof1, FM MK 2 & Prof3, IM ICS1 & PC2 & Prof3, PMR MK8 & Prof1, Rad ICS1, TY ICS 1, 3 & Prof1 |

| **Individualizes care by engaging patients in shared decision making and motivating behavior change** | | | | |
| --- | --- | --- | --- | --- |
| **Milestones** | | | | |
| **Level 1** | **Level 2** | **Level 3** | **Level 4** | **Level 5** |
| Uses directive style of guiding patient decisions | Respects patient autonomy in healthcare decisions  Discusses potential challenges associated with integrating patient values and beliefs with those of self, society, and the core values of medicine | Recognizes opportunities for shared decision making | Engages patients in shared decision making | Develops care plans jointly with patients and caregivers |
| Uses technical terms and jargon |  | Uses easy-to-understand language in all phases of communication | Assesses patient understanding of health information and invites questions | Consistently demonstrates communication strategies to ensure patient understanding and matches communication modality to the patient needs, health literacy and context |
| Conducts interview without inquiring about social and behavioral factors that affect the health of individuals | Identifies social and behavioral factors that affect the health of individuals and may be modifiable | Describes effective approaches to modifying individual health behaviors, such as shared decision making aids, motivational interviewing and health behavior coaching | Implements decision aids, motivational interviewing and/or health behavior coaching to modify individual health behaviors for patients who are motivated to change | Adapts decision aids and health behavior coaching techniques for patients who are not motivated to change |
| ^a^**Representative Corresponding ACGME Milestones**[23] | | | | |
| Derm ICS1 | Aero Med MK1, FM ICS1, TY Prof2 | Aero Med MK1, Occ Med MK1, Prev MK1, Psych ICS2, Derm ICS1, FM ICS2 | Aero Med MK1, FM ICS2, Oph ICS1,Occ Med MK1, Prev MK1, Psych ICS2, Uro ICS2, IM ICS1 | Aero Med MK1, FM ICS2, Neuro ICS, Occ Med MK1, Peds SBP1, Prev MK1, Psych ICS2 & Prof1,  PS Prof1 |
| **Practices contextual and cultural humility, curiosity, and awareness** | | | | |
| **Milestones** | | | | |
| **Level 1** | **Level 2** | **Level 3** | **Level 4** | **Level 5** |
| Does not demonstrate an interest in patients as individuals nor in the beliefs, values, and practices of diverse patient populations  Shows limited awareness of differences related to age, gender, race, culture, ethnicity, sexual orientation, religion, socioeconomic status, etc. in patient/caregiver encounters  Demonstrates disrespect, indifference or insensitivity towards patients | Demonstrates an interest in patients as individuals and in the beliefs, values, and practices of diverse patient populations  Is sensitive to differences related to age, gender, race, culture, ethnicity, sexual orientation, religion, socioeconomic status, etc. in patient/caregiver encounters  Treats patients with dignity, sensitivity, and compassion (regardless of race, culture, gender, ethnicity, age, sexual orientation, religion, socioeconomic status, etc.) in uncomplicated situations | Describes strategies for eliciting sociocultural factors that could impact health and health behaviors from patients and caregivers  Speaks about patients in a respectful and sensitive way when the patient is present | Elicits sociocultural factors that could impact health and health behaviors from patients and caregivers  Speaks about patients in a respectful and sensitive way even when the patient is not present  Treats patients with dignity, sensitivity, and compassion (regardless of race, culture, gender, ethnicity, age, sexual orientation, religion, socioeconomic status, etc.) in complicated situations | Adapts interactions with patients and families to the sociocultural context    Encourages others to speak about patients in a respectful and sensitive way even when the patient is not present  Encourages others to treat all patients with dignity, sensitivity, and compassion (regardless of age, gender, race, culture, ethnicity, sexual orientation, religion, socioeconomic status, etc.) |
| Cannot identify situations requiring interpreter services | Identifies situations requiring interpreter services, yet requires assistance to access and use interpreters | Describes best practices for using an interpreter and discusses potential challenges related to using interpreters | Independently accesses and uses interpreters | Appropriately uses interpreters to facilitate communication with patients and caregivers |
| ^a^**Representative Corresponding ACGME Milestones**[23] | | | | |
| IM Prof3 | IM Prof 3 | FM Prof3, PS ICS | Derm Prof3, TY Prof1 | Derm ICS1, IM Prof1&3 |

| **Develop skills in advocacy theory, execution and communication in order to advocate effectively for individual patients and at-risk patient populations** | | | | |
| --- | --- | --- | --- | --- |
| **Milestones** | | | | |
| **Level 1** | **Level 2** | **Level 3** | **Level 4** | **Level 5** |
| Cannot define advocacy for individual patients and/or at risk patient populations nor provide examples | Defines advocacy and for individual patients and at-risk patient populations and provides examples | Identifies and defines the scope of problems amenable to advocacy (the spectrum of physician advocacy) | Recognizes opportunities to advocate on the behalf of individual patients and for populations | Effectively advocates for individual patient(s) and/or at-risk populations to improve care |
| Does not recognize that physicians have a role in advocacy | Recognizes the role of physicians in advocacy | Explains how advocacy benefits patient care | Identifies different care team members as a resource to assist in patient advocacy | Utilizes interdisciplinary and system resources to advocate for individual patients and at-risk populations |
| Does not recognize the responsibility to assist patients and families in the navigation of the complex health care system | Recognizes the responsibility to assist patients and families in navigation of the complex health care system | Actively assists patients and families in navigating the complex health care system | Recognizes complexities of the health care system that make navigation difficult for at-risk populations | Advocates for system changes to improve at-risk populations in navigating the health care system. |
| ^a^**Representative Corresponding ACGME Milestones**[23] | | | | |
|  | Prev SBP3, Psych MK6 | Peds SBP1, Psych MK6 | IM Prof1, Neuro Prof 1 | Peds SBP1, IM SBP3, Prev SBP3, Psych SBP2 |

| **Partners with the community to improve individual and population health** | | | | |
| --- | --- | --- | --- | --- |
| **Milestones** | | | | |
| **Level 1** | **Level 2** | **Level 3** | **Level 4** | **Level 5** |
| Lacks awareness of impact of community on individual and population health | Recognizes that physicians can impact community health  Understands basic epidemiologic concepts and recognizes that diseases and injuries occur in a social and environmental context  Lists ways in which community characteristics and resources affect the health of patients and communities  Identifies common health issues in a community | Identifies community resources for patients and families  Identifies broad social or environmental factors that may impact the health of a community  Selects and describes appropriate health status measures to assess a community’s health | Engages community partners to educate a community or group  Mobilizes team members and links patients with community resources to achieve health promotion and chronic disease management goals  Collaborates with community resources to help manage patients’, families’, and population’s needs and conditions  Understands the process of conducting a community strengths and needs assessment | Conveys complex health information to educate a community or group and responds to queries about risk  Participates in creating new community based programs.  Monitors and interprets multiple or complex health status indicators of the community |
| ^a^**Representative Corresponding ACGME Milestones**[23] | | | | |
|  | FM SBP3, Prev MK2 & PC 2&8, Pysch SBP2, Psych Prof2 | Aero Med MK2 & PC2, FM SBP3, Occ Med MK2, Prev PC2 | FM ICS2 & PC2, Pysch SBP3 | Aero Med PC2, FM PC3, Occ Med PC4&5, Pysch SBP3 |

| **Applies principles of preventive health and strategies for population health management** | | | | |
| --- | --- | --- | --- | --- |
| **Milestones** | | | | |
| **Level 1** | **Level 2** | **Level 3** | **Level 4** | **Level 5** |
| Lacks knowledge of prevention as a method to impact health of individuals and communities | Explains the basis of health promotion and disease prevention  Defines primary, secondary, and tertiary preventive approaches to disease prevention and health promotion for individual or communities | Demonstrates awareness of health maintenance and screening guidelines and how to access them | Identifies appropriate preventive services for individual patients  Identifies preventive service needs for specific populations of patients | Educates and counsels individual patients on preventive services and health promotion with consideration of risks, benefits, and costs  Engages in population  -based strategies to enhance preventive services among populations of patients |
| Does not recognize distinctions between population and individual health services  Lacks knowledge of strategies to measure and manage the health of populations | Identifies various individual and population-based health services delivery settings and systems  Defines variables relevant to population health management in a dataset | Uses literature to identify a target population for a given population-based health service  Identifies a population health question that can be answered using a dataset | Answers a question regarding population health management using dataset | Applies results of analysis of population health dataset to an individual or populations of patients |
| ^a^**Representative Corresponding ACGME Milestones**[23] | | | | |
| Prev PC5 | Aero Med PC5, Derm Prof3, FM PBLI3 & PC2, Occ Med SBP1, Prev SBP1 | Derm SBP1, FM PC3, Occ Med MK4, Peds PBLI3, Prev PC5, Psych SBP3 | Aero Med MK4 & PC5, FM PC3, Occ Med PC7,PMR PC4, Psych SBP3 | Aero Med MK4 & PC4&5, FM ICS2 & PC3, Occ Med SBP2 & PC4,6&7, Prev PC4 |

| **Collaborates as a member of an interprofessional team and demonstrates effective, team-based patient care** | | | | |
| --- | --- | --- | --- | --- |
| **Milestones** | | | | |
| **Level 1** | **Level 2** | **Level 3** | **Level 4** | **Level 5** |
| Disrespectful in interactions with patients, caregivers, and members of the interprofessional team  Fails to recognize members of the interdisciplinary team as being important  Resists offers of collaborative input and refuses to recognize the contributions of other team members  Ignores feedback and is unwilling to change behavior to reduce the risk for errors | Recognizes that effective relationships are important to quality care  Describes own role as member of health care team  Explains how teamwork benefits patient care; recognizes failures in teamwork as a leading cause of preventable patient harm  Willing to receive feedback from members of the healthcare team | Aware of the unique contributions of other health care professionals and seeks their input for appropriate issues  Describes use of Crew Resource Management or similar communication techniques for patient care teams  Reflects upon and learns from own clinical incidents that may lead to medical error | Independently develops and sustains relationships with families and health care team members  Identifies situations when the breakdown in teamwork or communication may contribute to medical error  Solicits feedback from all members of the interprofessional team and patients | Sustains positive relationships with families and health care providers during challenging situations  Leads briefings and executes teamwork techniques designed to prevent adverse events  Consistently demonstrates the ability to identify limits of own knowledge and proactively solicits and incorporates feedback from all members of the interprofessional team and patients |
| ^a^**Representative Corresponding ACGME Milestones**[23] | | | | |
| IM SBP1&2 & ICS2 & Prof1, Peds SBP3 | Derm Prof2 & SBP2&4, FM ICS1, IM ICS2 & SBP1&2, Med Gen ICS, Oph SBP3, Ortho ICS,  Rad Onc ICS, TY SBP2,  Uro SBP1 | CS ICS, FM SBP4, IM SBP2, Oph SBP3, Path Prof4, Peds SBP3, PMR Prof1, Uro P2 | Derm ICS3, EM SBP1, Med Gen ICS, Occ Med Prof2, Ortho ICS, Path Prof4 | Derm ICS3, IM PBLI3, Path Prof4, PMR ICS1, Surg ICS2, TY ICS3, Uro SBP3 |

| **Effectively leads an intra-/ interprofessional team in the clinical, educational, or research settings** | | | | |
| --- | --- | --- | --- | --- |
| **Milestones** | | | | |
| **Level 1** | **Level 2** | **Level 3** | **Level 4** | **Level 5** |
| Disregards personality type and leadership style in role as a team member or leader  Dominates team or inappropriately assumes leadership role | Describes the variety of personality and leadership styles and identifies how his/her leadership style may impact intra- and inter-professional teams  Describes the fundamental skill set for effective leadership  Explains the concept of leading by example  Leads team of peers in classroom setting | Uses the knowledge of his/her leadership style or personality type to reflect on team interactions and leadership experiences  Discusses personal role as leader | Demonstrates good team leadership skills, including providing direction, inviting and utilizing input, providing feedback, creating a positive team climate, and managing conflict  Helps lead a multidisciplinary team  Recognizes duality of roles in that at times he or she must be able to step into a leadership role, while at other times must act as basic team member | Anticipates team needs and takes leadership role to independently implement solutions  Leads team –based care activities  Anticipates team dynamics and effectively manages interactions to optimize group performance |
| ^a^**Representative Corresponding ACGME Milestones**[23] | | | | |
|  | Derm Prof2 |  | PS SBP1, Uro ICS5, Surg ICS3, Psych SBP1 | Ortho ICS, Path Prof4  PMR SBP2, Psych ICS1 |

| **Effectively gives and receives a patient handover to transition care responsibility** | | | | |  |
| --- | --- | --- | --- | --- | --- |
| **Milestones** | | | | |  |
| **Level 1** | **Level 2** | **Level 3** | **Level 4** | **Level 5** |  |
| Disregards need for communication at time of transition  Conducts handoffs without considering patient privacy and confidentiality  Does not have a basic understanding of resources available for coordination of patient care | Outlines the importance of transitions in the continuum of care & recognizes the importance of communication during times of transition  Lists the risks inherent in handoffs  Lists/describes steps appropriate for care transition  Defines/describes SBAR (or similar tool) for handoffs in healthcare  Describes resources available for coordinating patient care | Applies structured communication techniques and tools during handoffs and changes in patient condition  Uses a standard template for the information provided during the hand-off; is unable to deviate from that template to adapt to more complex situations; makes errors of omission or commission  Lists potential impediments to safe and efficient transitions of care | Ensures transitions of care are accurately and efficiently communicated  Adapts and applies a standard template to increasingly complex situations in a broad variety of settings and disciplines; ensures open communication, whether in the receiver or the provider of information role  Effectively uses EHR to exchange information among the health care team  Identifies impediments to safe and efficient transitions of care | Role models and teaches effective transitions of care; adapts and applies the template without error and regardless of setting or complexity; internalizes the professional responsibility aspect of handoff communication  Proposes effective solutions to impediments to safe and efficient transitions of care  Demonstrates effective use of all forms of technology to optimize continuity of care of patients and transitions of care | |
| ^a^**Representative Corresponding ACGME Milestones**[23] | | | | |  |
| IM SBP4, Surg SBP1 | IM SBP4, Oph ICS2 &SBP3, Path SBP1, Surg SBP1, TY SBP1 | Peds PC3, Oph SBP1, Uro SBP3 | EM ICS2, FM ICS3, Oph SBP1, Peds PC3, Uro ICS4 | FM ICS4, IM SBP4, Uro ICS4, Med Gen, Oph SBP1, Psych SBP3 | |

| **Defines value in health care and applies high value care strategies for individual patients and populations** | | | | |
| --- | --- | --- | --- | --- |
| **Milestones** | | | | |
| **Level 1** | **Level 2** | **Level 3** | **Level 4** | **Level 5** |
| Cannot define value in health care  Ignores value issues (such as cost, safety, minimizing unnecessary tests and interventions) in the provision of care | Defines value in health care using the value equation, and explains how perceptions of value differ among stakeholders  Demonstrates general awareness of value issues in provision of care, but does not demonstrate application when caring for patients in clinical setting (e.g. when selecting tests, procedures, therapies) | Provides clinical examples of cost and value implications of care for individual patients    Demonstrates application of value issues when caring for patients in clinical setting (e.g. when selecting tests, procedures, therapies) | Provides clinical examples of cost and value implications of care for populations of patients  Conducts effective high-value cost conscious care conversation with individual patient in common clinical scenario  Meaningfully reflects on cost and value implications of care he or she provides | Selects diagnostic testing based on cost-effectiveness and likelihood that results will influence clinical management in common clinical scenarios |
| Does not identify barriers to high-value cost conscious care | Identifies patient-specific preferences for and barriers to high-value cost conscious care | Identifies system-level opportunities to improve outcomes, minimize harms and/or reduce health care waste | Utilizes resources and tools to identify opportunities for high-value cost conscious care for a population of patients | Meaningfully participates in activity that closes gap in delivery of high-value cost conscious care for a population of patients |
|  |  |  |  |  |
| ^a^**Representative Corresponding ACGME Milestones**[23] | | | | |
| IM SBP3 | IM SBP3 | Derm SBP4, IM SBP2, TY SBP3 | IM SBP3, Ortho | Psych SBP2, PMR PC3 |

| **Identifies systems failures and errors and contributes to a culture of safety and quality improvement** | | | | |
| --- | --- | --- | --- | --- |
| **Milestones** | | | | |
| **Level 1** | **Level 2** | **Level 3** | **Level 4** | **Level 5** |
| Cannot define common terms used in patient safety or identify patient safety events  Cannot differentiate between errors attributable to systems and individual errors  Lacks knowledge of standardized tools to analyze medical error (such as a fishbone diagram) | Defines common terms used in patient safety (such as types of safety events, levels of harm)  Identifies and classifies patient safety events in clinical case examples  Distinguishes systems errors from individual errors | Identifies and classifies patient safety events in clinical care  Does not demonstrate awareness regarding need for teams and/or self to analyze clinical performance to limit errors and quality gaps  Uses a standardized tool (such as a fishbone diagram) to analyze medical error case, but does not suggest viable interventions to prevent similar errors. | Reports patient safety events and/or seeks to contribute to system improvement  Advocates for just culture/culture of safety in clinical setting  Demonstrates awareness regarding need for teams and self to analyze clinical performance to limit errors and quality gaps  Uses a standardized took to analyze medical error case, and suggests viable interventions to prevent similar errors | Leads other team members in reporting patient safety events and/or seeking improvement  Teaches others about culture of safety  Recognizes and volunteers responsibility for decisions that may create quality gaps/increase risk for error, and takes steps to change behavior  Leads a team which analyzes a clinical medical error and suggests viable interventions to prevent similar errors |
| ^a^**Representative Corresponding ACGME Milestones**[23] | | | | |
| IM PBLI2 & SBP2 | NS SBP2 | IM SBP2, Occ Med PBLI1 & SBP3,  Oph ICS2 & PBLI3, Path SBP4, PMR PBLI 3, Prev SBP3 | Derm SBP3, IM PBLI2,  NS, Nuc Med, Oph PBLI3 & SBP3, Ortho SBP3, Prev SBP3, Psych SBP1, Surg SBP2, TY PBLI3, Uro SBP1 | Derm Prof2, FM SBP2, IM SPB2, Nuc Med, Occ Med PC13 & PBLI1, Oph ICS3,  Path Prof2 & PBLI1 & SBP4, Prev SBP1, Surg Prof1 & SBP2 |
| **Forms clinical questions and retrieves, appraises and assimilates evidence from the scientific literature** | | | | |
| **Milestones** | | | | |
| **Level 1** | **Level 2** | **Level 3** | **Level 4** | **Level 5** |
| Does not recognize and/or acknowledge need to seek out new information in the context of clinical scenarios even with prompting | Understands the need to seek out new information in the context of clinical scenarios with prompting | Seeks new clinical information in the setting of clinical settings with prompting  Can translate medical information needs into clinical questions with assistance | Reliably identifies the need to seek out new information in the context of clinical settings without significant prompting  Formulates searchable questions from clinical questions and uses informational technology to answer them | Consistently identifies, obtains and shares new information for team members in the context of clinical settings  Takes ownership and recognizes need to lead team members in use of evidence based care for patients |
| Considers all evidence to be of equal weight | Describes the ‘hierarchy of evidence’ concept | Uses medical information systems to information search but lacks ability to discriminate resources and search efficiently  Demonstrates early skill in appraisal of sources and content of medical information but requires assistance | Demonstrates the ability to select and search appropriate evidence-based information tools to answer specific clinical questions but unable to do so efficiently at point of care  Can categorize and rank study design and look at validity of studies including bias and confounding factors with minimal assistance | Able to confidently select an appropriate evidence-based information tool and to use search filters to hone in on the best answers to specific questions at point of care  Ranks study design and can distinguish relevant research outcomes (e.g., patient-oriented evidence that matters) from other types of evidence |
|  |  |  |  |  |
|  |  |  |  |  |
|  |  |  |  |  |
| ^a^**Representative Corresponding ACGME Milestones**[23] | | | | |
|  | IM PBLI4, Uro PBLI4 | IM PBLI4, Oph PBLI1,  Uro PBLI3 | Neuro PBLI, Oph PBLI2, Ortho PBLI, PMR PBLI2, Prev PC10, Uro PBLI2 | IM PBLI4, Ortho PBLI, PS PBLI, TY PBLI2, Uro PBLI4 |

| **Describes United States’ healthcare financing and related effects on patient care and quality** | | | | |
| --- | --- | --- | --- | --- |
| **Milestones** | | | | |
| **Level 1** | **Level 2** | **Level 3** | **Level 4** | **Level 5** |
| Does not demonstrate awareness of U.S. health payment systems  Incorrectly identifies roles of relevant stakeholders in U.S. health care payment systems | Describes U.S. health payment systems, including uninsured  Describes relevance of macroeconomic effect of health care on the rest of the U.S. economy | Describes advantages and disadvantages of different payment systems, types of payers, and mechanisms of reimbursement  Differentiates economics of inpatient vs. outpatient care | Identifies impact of quality improvement incentives and cost impact of practice variation  Identifies roles and varied impacts on cost and quality of care for the following stakeholders: providers, commercial and government payers, pharmaceutical and device companies, health care systems, hospitals, insurance carriers | Determines how different types of payment systems can influence a particular patient’s utilization of and access to health care  Debates current issues of health care financing and how they may affect providers, patients, third party payers, and other stakeholders |
| ^a^**Representative Corresponding ACGME Milestones**[23] | | | | |
|  | NS SBP, PS SBP | Nuc Med SBP, Rad SBP2 | Derm SBP4, NS SBP, Nuc Med SBP, Uro SBP2 | Derm SBP4 |

| **Analyzes current United States’ healthcare policy and its impact on health care delivery systems** | | | | |
| --- | --- | --- | --- | --- |
| **Milestones** | | | | |
| **Level 1** | **Level 2** | **Level 3** | **Level 4** | **Level 5** |
| Cannot identify systems of care  Does not demonstrate awareness of current health care policies | Describes basic levels of systems of care  Identifies the types of health care providers within a health care delivery system  Discusses current state and national health care policies and their implications | Recognizes various individual and population-based health care services delivery settings and systems  Recognizes how health care systems influence individual practice and patient care  Discusses advocacy and policy development | Works and coordinates patient care in various delivery systems for common clinical situations  Works within the institution to develop hospital systems that enhance safe patient disposition and maximize resource utilization  Assesses organizational performance of health care delivery system | Interacts with other stakeholders to improve the performance of the system  Uses evaluation findings to recommend strategic or operational improvements  Participates in advocacy activities for health policy |
| ^a^**Representative Corresponding ACGME Milestones**[23] | | | | |
|  | Nuc Med SBP, Oph SBP1, Uro SBP1 | Aero Med SBP1, Med Gen SBP, Psych MK6 | EM PC7, Occ Med SBP1,  Rad Onc | Aero Med PC5 & SBP1,  PS SBP |

| **Describe the role of clinical informatics, healthcare IT, and technology assessment in improving patient outcomes** | | | | |
| --- | --- | --- | --- | --- |
| **Milestones** | | | | |
| **Level 1** | **Level 2** | **Level 3** | **Level 4** | **Level 5** |
| Does not recognize ethical and legal implications of using technology in health care  Does not demonstrate awareness of role, risks, and limitations of Electronic Medical Record (EMR)  Disregards patient confidentiality | Recognizes ethical and legal implications of using technology in health care  Recognizes legal, financial, and patient care role of EMR  Recognizes risks and limitations added by EMRs  Describes legal rights of patients to confidentiality of personal health information as stipulated in Health Insurance Portability and Accountability Act | Identifies commonly used surveillance data sources (vital statistics, hospital discharge data) and conditions typically monitored using such systems  Explains role of EMR and CPOE in prevention of medical errors | Uses decision support systems in EMR (as applicable in the institution)  Applies available technology to optimize transitions of care and prevent medical errors | Proposes development of changes in policies and procedures related to technology to impact patient outcomes  Recommends systems re-design for improved technology and media processes |
| ^a^**Representative Corresponding ACGME Milestones**[23] | | | | |
|  | FM C4, Occ Med PC8, TY ICS4, Uro SBP4 | Prev PC9, Uro SBP4 | FM ICS4, Med Gen SBP, Uro SBP4 | EM SBP3, Psych ICS2 |

**^a^**Referenced milestones are representative, not comprehensive. Reference Milestone Abbreviations: ACGME = Accreditation Council of Graduate Medical Education, EMR = electronic medical record, ICS = interpersonal and communication skills, IT = information technology, MK = medical knowledge, PBLI = practice-based learning and improvement, PC = patient care, Prof = professionalism, SBP= systems-based practice. Specialty Abbreviations: Aero Med = aerospace medicine, CS= colon and rectal surgery, Derm = dermatology, Rad = diagnostic radiology, EM = emergency medicine, FM = family medicine, Surg = general surgery, IM = internal medicine, Med Gen = medical genetics, NS = neurosurgery, Neuro = neurology, Nuc Med = nuclear medicine, Occ Med = occupational medicine, Oph = ophthalmology, Ortho = orthopedic surgery, Path = pathology, Peds = pediatrics, PS = plastic surgery, PMR= physical medicine and rehabilitation, Prev = preventative medicine and public health, Psych = psychiatry, Rad Onc = radiation oncology, TY = transitional year, Uro = urology
